# The microRNA-183 cluster: the family that plays together stays together

**DOI:** 10.1093/nar/gkv703

**Published:** 2015-07-13

**Authors:** Shweta Dambal, Mit Shah, Brittany Mihelich, Larisa Nonn

**Affiliations:** 1 Department of Pathology, University of Illinois at Chicago, 840 S. Wood Street, Room 130 CSN, MC 847, Chicago, IL 60612, USA; 2 University of Illinois Cancer Center, Chicago, IL 60612, USA

## Abstract

The microRNA (miR)183 cluster, which is comprised of miRs-183, -96 and -182, is also a miR family with sequence homology. Despite the strong similarity in the sequences of these miRs, minute differences in their seed sequences result in both overlapping and distinct messenger RNA targets, which are often within the same pathway. These miRs have tightly synchronized expression during development and are required for maturation of sensory organs. In comparison to their defined role in normal development, the miR-183 family is frequently highly expressed in a variety of non-sensory diseases, including cancer, neurological and auto-immune disorders. Here, we discuss the conservation of the miR-183 cluster and the functional role of this miR family in normal development and diseases. We also describe the regulation of vital cellular pathways by coordinated expression of these miR siblings. This comprehensive review sheds light on the likely reasons why the genomic organization and seeming redundancy of the miR-183 family cluster was conserved through 600 million years of evolution.

## INTRODUCTION

In the past decade, microRNAs (miRs) have emerged as important players in RNA interference-mediated post-transcriptional gene regulation. The canonical role of miRs is to bind the 3′ untranslated region (UTR) of target messenger RNAs (mRNAs) via a complementary nucleotide seed sequence to reduce mRNA stability and/or suppress protein translation (1). miRs may be transcribed from individual genes or as clusters (2). A cluster of miRs is defined as several miR genes located adjacent to each other on the chromosome, which are transcribed as one long primary miR (pri-miR) transcript and then processed into the individual precursor miRs (pre-miRs) (3). The genomic organization of miRs in a cluster may serve to protect it from degradation as the secondary structure of a longer pri-miR is complex with numerous hairpins that stabilize the RNA (4). miR clusters range from <100 base pairs (bp) to 50 kilobases (kb) (3,4), and are often transcribed by a common promoter (5,6). miRs within a cluster are often, but not always, paralogous with high sequence homology, indicating that they may be the result of genomic duplications (7,8). High sequence homology between the miRs in a cluster classifies them as a family and permits both common and unique mRNA targets. Frequently, these mRNA targets lie within the same pathway, thereby allowing these miRs to have regulatory control over several components of a cellular process. Consistent with this role for miR clusters, several clusters have been found to be essential for normal development and disease pathology (9–17). For example, miR-17–92, one of the most extensively studied miR clusters, is necessary for normal skeletal development and was the first human miR oncogene or ‘oncomiR’ identified (6,18–21).

In this review, we discuss the characteristics and functions of the highly conserved miR-183 cluster, which is comprised of paralogous miRs-183, -96 and -182 (22,23). We begin with the discovery of the miR-183 cluster, its genomic organization and conservation. Normal functions of these miRs in development as well as dysregulation of the members of the miR-183 family in disease are discussed. Lastly, we integrate the targets of these miRs and the transcription factors that regulate the miRs into pathways and discuss regulation of vital processes by the miR-183 family members.

### Discovery of the miR-183 cluster and structural organization

**Figure 1.**
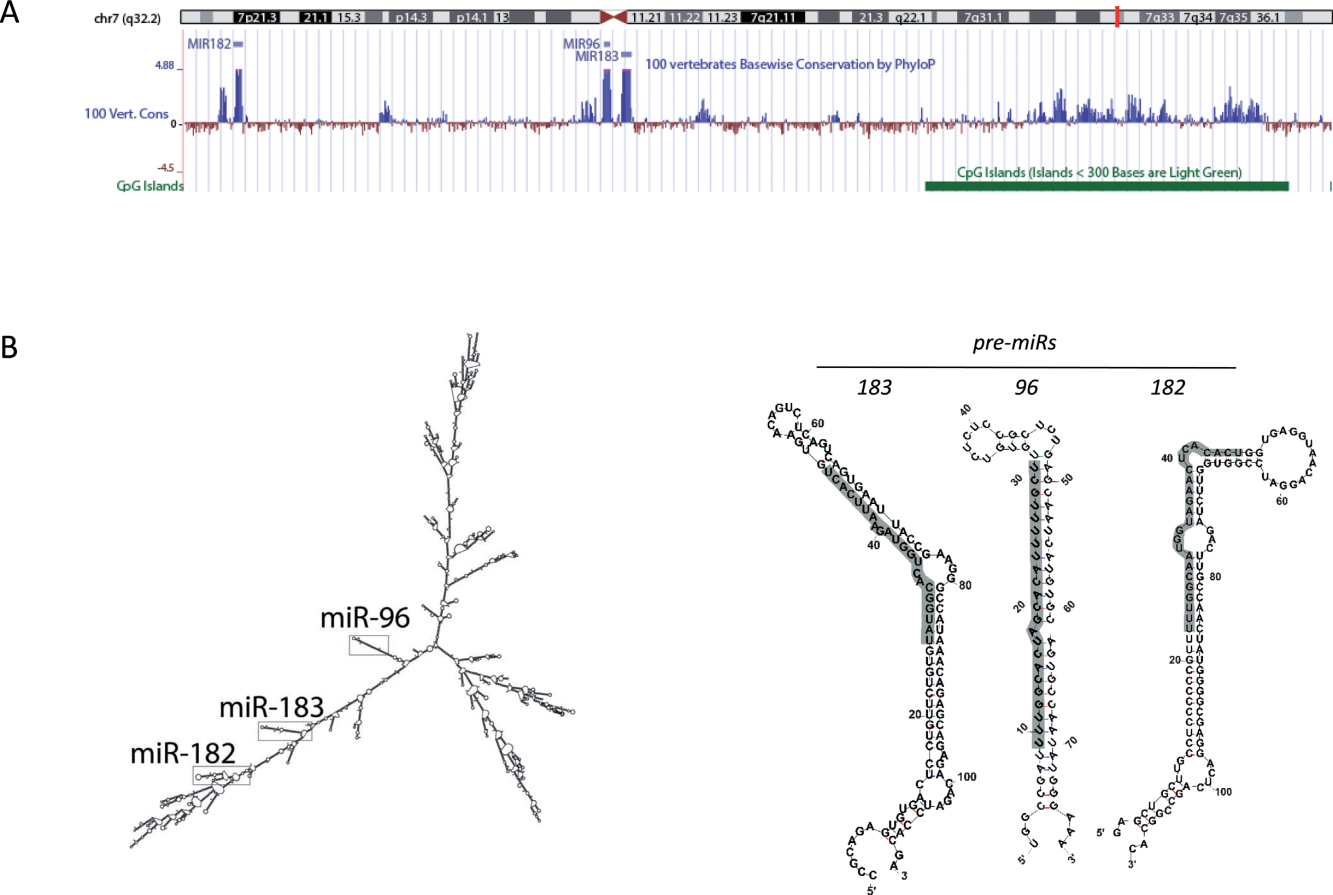
Genomic organization and structure of the human miR-183 cluster. (**A**) University of California, Santa Cruz (UCSC) genome browser (170) snapshot of the hsa-miR-183 cluster (miRs-183, -96, -182) on assembly Human Feb. 2009 (GRCh37/hg19). (**B**) Secondary structures of hsa-pri-miR-183–96–182, hsa-pre-miR-183, -96 and -182 as predicted by Mfold RNA folding software (29). The mature 5′ miR sequence is highlighted in grey.

The first human miR of the cluster to be identified was miR-96, which was immunoprecipitated along with ribosomes in a screen for ribosome-interacting small RNAs in the HeLa human cancer cell line (24). miRs-182 and -183 were identified in 2003 by separate groups; Lim *et al*. identified the miRs via bioinformatic comparison of human and mouse RNAs to the genome of *Fugu rubripes* (Japanese pufferfish) (25) and Lagos-Quintana *et al*. observed these miRs as highly expressed in the murine retina during development (26). miRs-182 and -183 were classified as a cluster due to their co-expression in retina and chromosomal proximity to each other (26). miR-96 was subsequently determined to be part of this cluster due to its sequence homology to and chromosomal location between miRs-183 and -182 (27,28) (Figure 1A).

**Figure 2.**
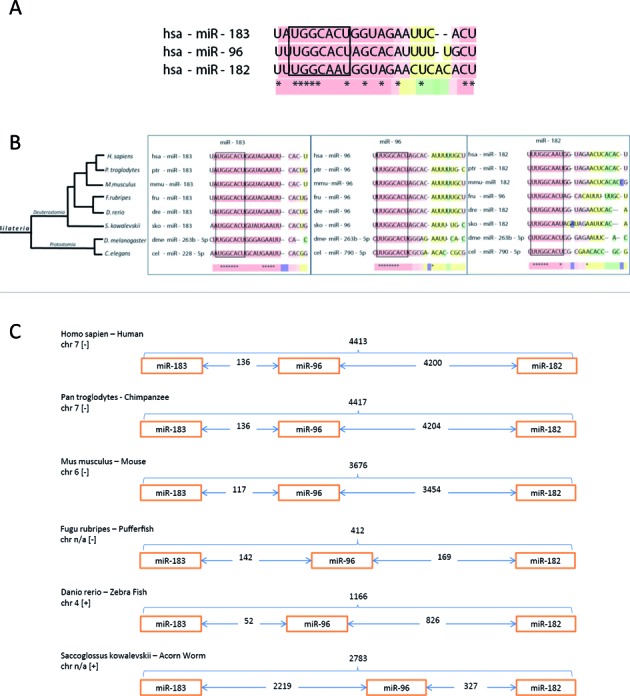
Conservation of the miR-183 cluster over 600 million years. (**A**) Sequence homology of the hsa-miR-183 family. (**B**) Phylogenetic tree of the miR-183 family created using the NCBI taxonomy browser (36,37). Sequences for the miRs were obtained from MirMaid (38) and miRbase (40–44). Tcoffee alignment software (39) was used to generate the sequence homology image. (**C**) Intergenic spacing of the miR-183 cluster using chromosomal location data obtained from miRBase.

The polycistronic nature of the miR-183 cluster was demonstrated by 5′ Rapid Amplification of cDNA Ends (RACE) of the murine pri-miR-183 cluster transcript in murine retina (27). The secondary structure of the( *Homo sapiens* (hsa)-pri-miR-183–96–182 [using Mfold RNA folding software (29–31)] places the three pre-miRs in close proximity despite the >4 kb span between pre-miRs-96 and -182 (Figure 1B). The pri-miR-183–96–182 structure also contains multiple other hairpins which may increase RNA stability (4). The three trimmed pre-miRs (Figure 1B) are structurally different, though the mature miRs-183, -96 and -182 have near identical seed sequences (Figure 2A). A single base difference in the seed sequence of miRs-96 and -182 is essential for its mRNA target specificity as shown by differential regulation of glypican-1 and glypican-3 mRNA (32,33). There is at least one more non-conserved paralog in the miR-183 family, miR-1271, which is located on human chromosome 5q35 and has mRNA targets similar to miR-96 (34).

### Conservation of the miR-183 cluster

The sequence homology of miRs-183, - 96 and -182 and the conservation of their genomic organization as a cluster in bilaterian organisms indicates an evolutionary advantage to retaining this microRNA cluster (Figure 2). Next-generation deep sequencing and comparative genomics have revealed that the miR-183 cluster can be evolutionarily traced back ∼600 million years ago to protostomes and deuterostomes (22,35–39) (Figure 2B). Although the chromosomal order of miR-183, -96, -182 is conserved in deuterostomes, their location and the intergenic spacing between the miR genes vary between species (Figure 2C, (40–44). In humans the cluster is located on chromosome 7, with a 4.2 kb intergenic region between miRs-96 and -182. However, the murine miR-183 cluster is located on chromosome 6 with 3.6 kb between miRs-96 and -182. In lower organisms, the spacing between the miRs is shorter with the exception of the sea urchin in which the genomic region from miR-183 to -182 is the longest of all at 5122 bp. The conservation of this intergenic region between miRs -96 and -182 suggests that it contains important regulatory and/or structural elements.

### The miR-183 cluster in normal cells and development

The functions of the miR-183 family members in normal cells and during development have been described in specific organs and emphasize a role in sensory organ development (22,26,45–49). Expression of the miR-183 cluster (or orthologs) is necessary for sensory/ mechanosensory organ development in *Danio rerio* (zebrafish) (45,47), *Mus musculus* (mice) (48), Drosophila *melanogaster* (22,49) and *Caenorhabditis elegans* (22). The miR-183 family is expressed at high levels specifically in the nose, ear, retina and cranial and dorsal root ganglions of zebrafish embryos as early as 72 hours post fertilization (45) as detected by *in situ* hybridization. In the mouse embryo expression of the miR-183 family was highest in the eye (26). Another murine study showed miR-182 and -183 expression was confined to the placenta and day 9.5 embryo, respectively (50).

The miR-183 family is involved in auditory development (28). Spatial and temporal expression analyses in the murine auditory apparatus showed that the levels of the miR-183 family correlated with the functional maturation of the inner ear (51). miR-96 was further implicated in hearing in the diminuendo (DMDO) mouse model of deafness, in which a functional mutation in the seed sequence of miR-96 (A>T substitution) was identified (52,53). The DMDO mice have progressive hearing loss and obstructed development of inner/outer cochlea hair cells (53). Further supporting a role for this family in the ear, miRs-96 and miR-182 promote hair cell fate in the inner ear in zebrafish (47) and it is postulated that these miRs target mRNAs in a concerted manner in hair progenitor cells, thereby switching these cells into a differentiated hair cell fate (54). Moreover, miR-183 cluster null mice showed significant vestibular defects that resulted in abnormal gate (48).

There are high levels of the miR-183 family members in the murine olfactory bulb and the eye (55). The miRs are especially highly expressed in the retinal photoreceptors, bipolar and amacrine cells of normal adult mouse tissues (27) and these miRs are light-responsive in the retina (56). Mature miRs-183, -96 and -182 decayed rapidly during dark adaptation and transcription of the miRs increased during light adaptation (56). This flux in the levels of the miR-183 family members influenced neurotransmitter removal by direct regulation of the voltage dependent glutamate transporter, SLC1A1, thereby showing that light adaptation-dependent miR expression affects neuronal metabolism (56). In addition to the auditory phenotype described above, the miR-183 cluster null mice exhibit retinal defects and have progressive retinal degeneration (48).

The miR-183 family is expressed in normal human embryonic stem cells (ES) (57,58) and benign adult human prostate epithelial cells (59). In ES cells, temporal reduction of miR-183 family levels at day 10 was essential for epidermal differentiation to neuroectodermal precursors as this differentiation was blocked by overexpression in the miRs (58). In benign human prostate epithelial cells, the miR-183 family regulated zinc homeostasis via regulation of several zinc transporters, which is essential for normal prostate physiology as the healthy prostate accumulates zinc 10-fold higher than other organs (59).

#### Pathways regulated by the miR-183 family in normal cells

Regulation of circadian rhythm by the miR-183 family has been shown in multiple organisms (27,60–61) and may contribute to the conservation of these miRs through evolution (62). The individual miR-183 family members regulate several genes within this pathway to provide both redundant and complementary regulation of circadian rhythm. Diurnal changes in dme-miR-263a and -263b, *D. melanogaster* orthologs of the miR-183 cluster, occurred in wild type flies, but expression of these miRs was diminished in arrhythmic clock mutant *cyc^01^* (62). In the adult mouse retina, the levels of these miRs follow a circadian variation in their expression levels, with the miR levels being ∼2-fold higher during zeitberg time (ZT) 17 (midnight) compared to ZT 5 (noon time) (27). The putative promoter region of the miR-183 cluster has numerous predicted binding sites for transcription factors responsible for maintaining the circadian rhythm in the eye (27). *Clock, Mitf* and *Adcy6*, which are regulators of circadian rhythm, are luciferase-validated targets of miR-182 (*Clock, Mitf*) (63) and miR-96 (*Adcy6*) (27). In the zebrafish, miR-183 directly targeted e4 binding protein 4–6 (e4bp4–6) and arylalkylamine N-acetyltransferase 2 (aanat2), both of which regulate circadian rhythm (61). Temporal regulation of the miR-183 cluster was also shown by miR profiling in the rat pineal gland in which these miRs remarkably represented ∼42% of the entire microRNA population at ZT7 (midday) (60,61) and were very low at ZT19 (midnight), thus underscoring a significant role for these miRs in circadian regulation.

Other than the genes involved in circadian rhythm, there are few defined targets of the miR-183 family members in normal development. Although there are robust sensory phenotypes as a consequence of loss of the miR-183 family (47–48,52), the direct mRNA targets are not yet identified.

### The miR-183 cluster and human disease

Individual and multiple members of the miR-183 family are frequently identified by profiling studies as having aberrant expression in cancers and other diseases. In contrast to the seemingly sensory-specific role of these miRs in normal development discussed above, the miR-183 family does not appear to be expressed at high levels in most non-sensory adult tissues. However, high levels of the miR-183 cluster members have been observed in pathologic conditions of neurons, autoimmunity and a wide variety of malignancies. Research into the role of the miR-183 family in *in vitro* and *in vivo* models of these diseases has provided insight into pathways important for disease.

#### Auto-immune disorders

The miR-183 cluster members may contribute to the pathogenesis of systemic lupus erythematosus (SLE) (64,65) and two rare uveal auto-immune genetic syndromes (66). Three different mouse models of SLE (64) showed increased splenic levels of the miR-183 family in diseased mice. These miRs may be regulated by estrogen and relevant to the increased risk of SLE in women as female SLE mice and estrogen-treated orchidectomized mice had significantly higher levels of the miR-183 cluster compared to intact male SLE or placebo-treated orchidectomized mice (65). In two genetic auto-immune syndromes, there is a single nucleotide polymorphism (SNP) located in pre-miR-182, rs76481776 (C→T), that is significantly associated with both Beçhet and Vogt–Koyanagi–Harada (VKH) syndromes (66), although the functionality of the SNP in these diseases is unknown.

#### Neuronal disorders

Members of the miR-183 family have been shown to directly reduce neuropathic pain (67–69) and nerve injury (70) whereas these miRs are disease promoting in several neurodegenerative disorders (71,72). Intrathecal treatment of animals with miR-183 (73) or miR-96 (67) reduced pain in a spinal nerve ligation (SNL) rat model of neuropathic pain and nerve injury, in which the miR levels decreased in the dorsal root ganglion (DRG) following SNL (67,74). This effect was mediated by miR-dependent inhibition of the sodium channel, voltage-gated, type III, alpha subunit *(Nav1.3)* (67,73). In a rat model of osteoarthritic (OA) knee pain (68) the miR-183 family levels were reduced in diseased animals. On the other hand, miR-182 expression was rapidly increased and required to stimulate healing after nerve injury via decreased proliferation and migration of Schwann cells by directly targeting fibroblast growth factor 9 (FGF9) and neurotrimin (NTM) (70).

In contrast to their healing role in nerve injury and pain, these miRs may contribute to the symptoms associated with neurodegenerative diseases. Elevated axonal levels of miR-183 directly contributed to symptoms in a mouse model of a neurodegenerative disorder, spinal muscular atrophy (SMA), as treatment with miR-183 inhibitors improved survival and motor function in the SMA mice (71). Similarly, high levels of miR-96 and its paralog miR-1271 were present in frontal cortexes from patients with Multiple System Atrophy (MSA) and in mouse models of the disease (72), although the effect of miR inhibitors was not examined in these studies.

#### Psychiatric disorders

There are several associations between psychiatric disorders and single nucleotide polymorphisms (SNPs) in the miR-183 cluster or in the 3′ UTR of their target mRNAs. Genome Wide Association Studies (GWAS) showed an association between the rs13212041 sequence variant of the serotonin receptor 1b (HTR1B) and attention-deficit hyperactivity disorder (ADHD) (75). This variant is in the 3′ UTR of HTR1B and leads to decreased miR-96 binding and regulation (76,77). A subsequent case control study identified an association of two other SNPs within the miR-183 cluster and ADHD without substance use disorders (75). The presence of these SNPs, rs2402959T/rs6965643A and rs2402959C/rs6965643G, gave an odds ratio of 1.25 (*P* = 0.037) and 1.36 (*P* = 0.024), respectively, in this large study with 695 ADHD adults and 485 sex-matched unrelated controls (75).

Depression is also associated with SNPs in miR-183 cluster. The T-allele of the rs76481776 that was associated with the genetically rare auto-immune disorders (66), is also associated with clinical depression and late insomnia in patients (63). This SNP has not yet been shown to be functional, but there is indirect evidence that it may reduce miR-182 activity, as increased expression of miR-182 mRNA targets was observed postmortem in isolated dentate gyrus granule cells from depressed patients with the rs76481776 polymorphism (78). Experimental rat studies also support that the miR-183 members may be involved in depression. In a rat model of behavioral depression, known as learned helpnessness, male rats that did not exhibit learned helpnessness had reduced levels of several miRs, including all of the miR-183 cluster members, suggesting that these miRs alter the transcriptional and molecular landscape during depression (79).

#### Cancer

Though global levels of mature miRs are typically decreased in malignant tissues (80), the miR-183 family members are often expressed at high levels in tumors, implying a gain of function benefit for these miRs during carcinogenesis. Further supporting an advantage to high levels of the miR-183 family to cancer cells, allelic imbalance, chromosomal breakpoints and copy number gains have been detected in the chromosomal region of the miR-183 cluster in cancers (81–85). While most of the phenotypes associated with high levels of the miR-183 family members were consistent with an oncogenic role, there are also some studies that show tumor suppressor activity, suggesting a context-and/or cell type-dependent phenotype for the miR-183 cluster in carcinogenesis (86). Here, we draw attention to examples regarding the miR-183 family members in brain, lung, hormonal (prostate and breast) and gastrointestinal cancers (summarized in Table 1).

**Table 1.** Expression of the miR-183 in development and disorders

	Development				Dysregulation in disease and disorders																			
					Cancer																Other			
miR	ES cells	Ear	Eye	Nose	Prostate	Breast	Lung	Osteosarcoma	Liver	Brain	Bladder	Retinoblastoma	Colorectal	Pancreatic	Ovarian	Endometrial	Gastric	Thyroid	Uveal Melanoma	Cervical	Psychiatric	Depression	Autoimmune	Nerve Pain
183	*	**	**	*	↑	↕	↕	↓	↑	↑	↑	↕	↑	↑	↕	↑	↑	↑	ND	↑	ND	ND	↑	↓
182	*	**	**	*	↑	↑	↕	ND	↑	↑	↑	↑	↑	↑	↑	↑	↕	↑	↓	↑	SNP	SNP	↑ SNP	↓
96	*	**	**	*	↑	↑	↑	ND	↑	↑	↑	↑	↑	↓	↑	↑	↑	↑	ND	ND	SNP	?	↑	↓

* = expressed at high levels and role uncharacterized, ** = required for normal development, ↑ ↓ = expression compared to healthy tissues, SNP = single nucleotide polymorphism associated with disease, ND = not determined.

The miR-183 cluster members consistently behave as oncomiRs and elevated levels of the miRs have been observed in gliomas (87–90) and medulloblastomas (91,92). Several studies in medulloblastoma show a connection between the miR-183 family and the sonic hedgehog (SHH) signaling pathway, which is involved in embryogenesis, and dysregulated in medulloblastomas. Ectopic overexpression of all three miRs in cultured medulloblastoma cells from the Ptch1^+/−^/Pten^Floxp/+^ transgenic mice (Ptch1 is the receptor for SHH), increased proliferation (93). A relationship between the miRs and SHH was seen in medulloblastoma patients, in whom expression of the miR-183 family members was higher in SHH-negative compared to SHH-positive tumors (94).

There are data to support an oncomiRic role for the cluster in lung cancer. The majority of studies in lung cancer cell lines show that both miRs-96 and -182 target tumor suppressor genes and promote cell proliferation, colony formation, migration and invasion (95–100). These miRs are also detectable in the sera of lung cancer patients and may potentially serve as prognostic and/or diagnostic markers (100). There have been two studies that find tumor-suppressor activities of these miRs in aggressive lung cancer cell lines. Yang *et al*. showed that knockdown of miR-182 in H1299 lung cancer cell line increased invasion, migration and metastatic activity of these cells via decreased levels of FOXO3 and N-cadherin. (98). Overexpression of miR-183 inhibited invasion and migration in 801D lung cancer cells and regulated metastasis genes (101). Again this emphasizes that the role of the miRs often varies between cell lines, likely due to differences in the transcriptional landscapes.

All members of the miR-183 cluster are expressed at high levels in hormonal cancers of the prostate, breast and ovary, compared to benign tissues (59,102–108). High levels of miR-182 may provide a growth advantage to prostate tumor cells as high levels of miR-182 in patient prostate tumors correlated with poor survival (103), and miR-182 directly targeted the metastasis suppressor 1 (MIM) to increase proliferation of prostate (103) and breast cancer cells (109,110). In breast and ovarian cancers, the role of miR-183 has not been consistent. In normal breast tissue, estrogen treatment increased miR-183 levels (111), whereas paradoxically the miR levels were highest in ER-negative breast cancers (106). As well, overexpression of miR-183 in breast cancer cell lines that already have high levels of the miRs, inhibited cell migration in T47D cells (106), akin to the suppressive effect of high miR-183 in lung cancer metastasis (101). The data regarding the role of the miR-183 family in ovarian cancer cells also indicates context-dependency as *in vivo* ovarian cancer cell xenograft growth and metastasis were reduced by anti-miR-182 expression (112), whereas ectopically expressed miR-183 inhibited migration, invasion and viability of ovarian cancer cell lines *in vitro* (113).

The miR-183 family is highly expressed in colorectal and hepatocellular carcinonomas (HCC) and typically has oncomiR activity via inhibition of mRNAs for tumor suppressor genes (114–119). Overexpression and knockdown experiments with individual miR-183 family members have been shown to promote, migration, proliferation and metastasis in colon cancer and HCC cells, phenotypes which are fundamental to carcinogenesis (116,120–122). miR-182 and -183 levels were 2-fold higher in colon cancer tissue from African Americans compared to Caucasian tumor tissue (123), and therefore may be relevant to health disparities, although this remains to be explored.

The role for the miR-183 family in gastric and pancreatic cancers is not clear as they have been shown to be both lower (124–126) and higher in tumors compared to normal tissues (127,128). Similarly, opposite phenotypes were observed *in vitro*, in which overexpression of miR-182 had both tumor suppressor-like actions (124–126) and oncomiR-like actions (127). There are also reports on the expression and function the miR-183 cluster members in other cancers including uveal melanoma (129), retinoblastoma (130,131), sarcomas (132,133), thyroid (134), cervical (135) and endometrial cancers (136–139) that we have not reviewed here. The miR-183 family expression levels in these cancers are summarized in Table 1.

### Regulation of major cellular pathways in disease by the miR-183 family

**Figure 3.**
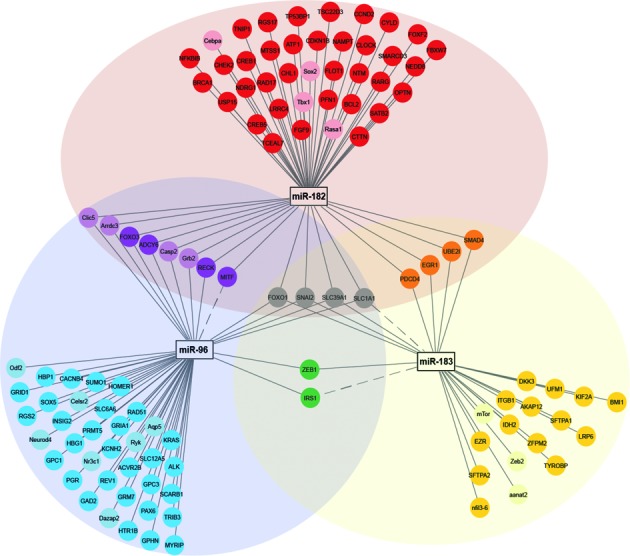
Network and Venn diagram of validated mRNA-targets of the miR-183 cluster shows overlapping and distinct messenger RNA targets. Network diagram was generated in Cytoscape 3.1. (140) Targets of miR-182 (red), miR-183 (yellow), miR-96 (blue), miRs-182 and -183 (orange), miRs-183 and -96 (green), miRs-96 and -182 (purple) and miRs-183, -182 and -96 (gray). Genes in lighter colors or dotted pathway lines represent miR-183 cluster messenger RNA targets in organisms other than humans.

**Figure 4.**
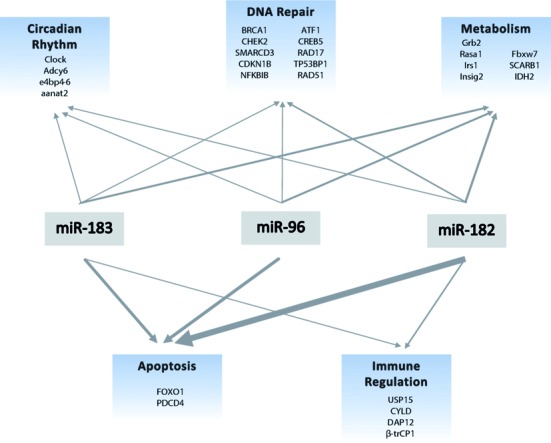
miR-183 cluster targets multiple cellular and biological pathways. Line weights are proportional to the number of literature reports that support individual miR or cluster involvement in above mentioned processes. miR-183 cluster targets are listed under appropriate pathways. References for each pathway can be found in Supplemental Table S2.

The miR-183 family may have been conserved through evolution to maintain regulation of vital processes as the redundancy in the binding sites may ensure regulation of their shared mRNA targets. Despite high sequence homology between these miRs, there are small differences in their sequences which have perhaps evolved over time to provide regulation of distinct mRNA targets often within the same functional group. To determine pathway cooperativity we performed detailed pathway enrichment analysis using only reporter gene-validated direct mRNA targets and the transcription factors that regulate them (Supplementary Table S1). Our analysis showed common and unique mRNA targets for the three miRs (140) (Figure 3). The mRNA targets of the miR-183 family and the miR-183 family-regulatory transcription factors concentrate in specific pathways (Figure 4, Supplemental Tables S1 and S2) (141), which are further described here.

#### Apoptosis

Most of the findings to date report the function of the miR-183 family is that of a regulator of apoptosis in cancer. Apoptosis, or programmed cell death, can be initiated by several intrinsic and extrinsic pathways, and is vital to maintaining healthy cell populations during or after exposure to physiological or pathological stimuli (142). The control over apoptosis often goes awry in disease (142) permitting survival of cells with damaged DNA. We discuss here how evidence in human cancer cell lines supports an anti-apoptotic role for the miR-183 family, whereas these miRs are pro-apoptotic in benign cells from organ systems such as the bone.

As mentioned in the previous section on cancer, high levels of the miR-183 cluster members are typically oncomiRic and anti-apoptotic (143–147). miR-96 inhibited apoptosis in both bladder cancer cells (T24) (145) and prostate cancer cells (LNCaP and DU145) (147) in which miR-96 was overexpressed. In both bladder and prostate cancer studies, apoptosis suppression was mediated by the negative regulation of FOXO1 by miR-96 (145,147). Pro-apoptotic programmed cell death 4 (PDCD4), was shown to be a shared target for both miR-183 and miR-182 in HCC cells, leading to chemoresistance to taxanes and cisplatin in ovarian cancer cells respectively (121,146). Inhibition of all three miRs in bladder cancer cells (T24 and UM-UC-3) and medulloblastoma cells (D458 and D556) increased the number of apoptotic cells and the expression of the pro-apoptotic markers BAD, BAK1 and BAX (91,148).

In contrast to the findings described above in cancer cells, miR-182 was pro-apoptotic in benign ovarian and bone cells. Overexpression of miR-182 in human primary ovarian granulosa cells enhanced apoptosis as evident by TUNEL and caspase-3 activation (149). Transient transfection of miR-182 in mesenchymal stem cells, MC3T3E1 pre-osteoblasts and primary calvaria cells, increased apoptosis and reduced proliferation (143). Furthermore, miR-182 suppression of FOXO1 mediated apoptosis in this study, demonstrating a dual role for FOXO1 being both pro- and anti-apoptotic (143). The pro-apoptotic effect of high levels of miR-183 family was also observed in the *D. melanogaster* retina, in which dme-miR-263a/b (orthologs of hsa-miR-183) inhibited the pro-apoptotic gene head involution defective (hid) and promoted cell survival (49).

The master apoptosis gate keeper, p53, may mediate the seemingly contradictory roles of the miR-183 family in apoptosis. The miR-183 cluster contains a p53 site in its promoter, which was ChIP-validated in primary benign human mammary cells (150). p53 function is lost in most malignancies (151) which may lead to dysregulation of miR-183 expression, thereby altering the miR-183 family-regulated post-transcriptional landscape during carcinogenesis.

#### DNA repair

In our pathway enrichment analysis, there were multiple DNA repair pathways with marked enrichment for miR-183 family target mRNAs, which may not be surprising given that this process is tightly associated with p53 and apoptosis (Supplementary Table S1). Here, we discuss miR regulation of DNA repair genes and how there may be a therapeutic advantage to high expression of the miR-183 family in tumors.

The miR-183 family impairs homologous DNA repair and high expression of these miRs enhanced the effectiveness of various cancer therapies. Of the three miRs of the cluster, miRs- 96 and -182 are the most established as negative regulators of mRNAs for DNA repair proteins (91,152–153). Ionizing radiation (IR) dose-dependently reduced the expression of miR-183 family members (153). In turn, the reduction of these miRs facilitated increased levels of nine mRNAs for proteins involved in homologous recombination (HR)-mediated DNA repair (CDKN1B, CHECK2, SMARCD3, NFKB1B, ATF1, CREB5, RAD17, TP53BP) (152) and RAD51 (154). miR-182 overexpression increased the amount of IR-induced double-strand breaks (DSBs) in HL60 leukemia cells (153). Conversely, knockdown of the entire cluster in medulloblastoma cells and knockdown of miR-182 in HL60 cells reduced DNA DSBs (91,153). Overexpression of miR-96 reduced HR-mediated DNA repair of IR-induced damage in human osteosarcoma U2OS cells (154). Overexpression of miR-96 with and without rescue of the mRNA targets showed that miR-96 mediated chemotherapy resistance to poly ADP ribose polymerase (PARP) inhibitors and cisplatin via targeting of *RAD51* and *REV1* (154). miR-96 also synergistically enhanced cisplatin sensitivity *in vivo* in MDA-MB-231 breast tumors, resulting in reduced tumor volume (154). *BRCA1* is a DNA repair-associated gene that, when mutated, contributes to familial breast cancer (155). *BRCA1* is a luciferase-validated target of miR-182 (152,153) and miR-182 overexpression sensitized breast cancer cells to PARP inhibitors *in vitro* and *in vivo* via inhibition of BRCA1 (153). However, in osteosarcoma cells (U2OS cells) overexpression of miR-182 did not affect IR-induced BRCA1 foci formation (154), providing another example of mRNA target heterogeneity in different cell types and transcriptional backgrounds.

#### Energy and metabolism

The miR-183 family regulates multiple mRNAs for proteins enriched in the citric acid (TCA) cycle, insulin signaling and lipid metabolism pathways. Thus, abnormally high levels of the miRs in disease have the potential to alter energy management and metabolism.

*In vitro* and clinical data support that miR-183 suppresses the TCA cycle by inhibition of the essential mitochondrial enzyme, isocitrate dehydrogenase 2 (IDH2) (89,156). IDH2 converts TCA cycle substrate isocitrate to α-ketoglutarate by oxidative decarboxylation. This permits a functional TCA cycle leading to chemical energy production by generating adenosine triphosphate (ATP) molecules. Negative regulation of IDH2 by miR-183 was suggested in clinical glioma tumor specimens in which *IDH2* (mRNA and protein) levels inversely correlated with miR-183 levels (89). This regulation was further demonstrated *in vitro* by overexpression of miR-183, which reduced *IDH2* leading to a reduction in α-ketoglutarate levels and triggered a hypoxic response (89). In A549 lung cancer cells and N12 primary normal lung fibroblasts high levels of CO_2_ increased miR-183 levels by 3.5-fold leading to suppressed *IDH2* levels, decreased mitochondrial oxygen consumption, reduced ATP production and inhibited cell proliferation (156). These *in vitro* findings may ultimately be clinically relevant as high CO_2_ (known as hypercapnia) manifests in patients with severe lung disorders including chronic obstructive pulmonary disorder (COPD), asthma, cystic fibrosis and muscular dystrophies.

The miR-183 family is integral to the regulation of lipid metabolism as they target mRNAs for lipid metabolism proteins and are themselves regulated by lipids. Jeon *et al*. proposed that this miR cluster serves as an ‘operon’ to regulate lipid metabolism. SREBP2-mediated transcription increased the hepatic levels of the miR-183 family ∼20-fold in mice treated with statins, lovastatin and ezetimibe, to reduce cholesterol (157). SREBP2 is a member of the Sterol Regulatory Element-Binding Protein (SREBP) family which regulate cell survival and lipid synthesis (158,159). SREBP2-induced miR-96 and miR-182 directly suppressed negative regulators of nuclear SREBP2 (insulin induced gene 2 (*Insig2*) and F-box/WD repeat-containing protein *(Fbxw7)* resulting in a positive feed forward loop to stimulate hepatic lipid synthesis and accumulation (157,160). Complementing this study, Wang *et. al* showed that miR-96 targeted hepatic scavenger receptor class B type (*SR-BI*) to reduce delivery of high-density lipoprotein cholesterol (HDL-C) for excretion and hormone synthesis to liver (161). Since HDL-C excretion has a protective effect in atherosclerosis, suppression of *SR-BI* by the miRs may increase risks for atherosclerosis and heart disease (161). Lastly, Xu and Wong computationally identified the miR-183 cluster as a regulator of the insulin signaling pathway in mice and validated growth factor receptor-bound protein 2 (*Grb2*), RAS p21 protein activator (*Rasa1*) and Insulin Receptor substrate 1 (*Irs1*) as mRNA targets of miR-96 and-182 (162). IRS1 and GRB2 bind the insulin receptor to initiate downstream RAS activation, thereby connecting insulin and energy metabolism to cell proliferation (162) similarly to SREBP2 (157).

#### Immune signaling

Immune signaling is a pathway enriched with miR-183 family mRNA targets and, as we described above, several auto-immune disorders have altered expression of these miRs. There is strong mechanistic data to show that these miRs directly target TGF-β and other cytokine signaling pathways (87,163–164). miRs-183 and -182 were both found to be transcriptionally up-regulated by TGF-β in separate studies (87,163). In glioma cells, TGF-β-induced miR-182 suppressed several NF-κB inhibitory proteins to facilitate constitutive NF-κB activity in glioma cells (87). Whereas TGF-β-induced miR-183 in natural killer (NK) cells directly suppressed *DAP12*, a signal adaptor for NK cell lytic function, ultimately leading to immunosuppression (163).

In T-cells the miR-183 cluster members regulate several pro-inflammatory cytokine pathways vital to the immune cell function (164,165). Expansion of T helper cells (T_H_) following interleukin-2 (IL2) stimulation required Stat5-mediated induction of miR-182, which subsequently suppressed FOXO1 (164). Of note, this is an example of differential regulation of the individual miRs as miR-182 expression was augmented after IL-2 treatment, and miR-96 was not detectable in the T_H_ cells (164). In U937 leukemia cells, IL-2 also increased miR-183 expression via a ChIP-validated Foxp3 binding site (165). Following *Schistosoma mansoni* infection in a rodent liver inflammation model, IL-4-induced differentiation of regulatory T (Treg) cells was mediated by an increase in miR-182 levels (165). This study indicates that miR-182 controls characteristics within Treg populations and may calibrate their function after exposure to specific pathogens (165).

An example of non-canonical miR binding within a coding region was shown with miR-183 and regulation of leukemia-associated inflammation (166). In U937 cells, miR-183 targeted and suppressed beta-transducin repeat containing E3 ubiqutin protein ligase (β-trCP) levels via binding to the coding region of the mRNA (166). β-trCP is a ubiquitin ligase (166) and reduction of β-trCP led to increased ADAM17 metalloproteinase and TNF-α shedding, which generated soluble TNF-α to further propagate inflammation (167).

### Regulation of miR-183 cluster expression

Throughout the literature there are examples of the miR-183 family members being transcriptionally regulated together as a single polycistronic pri-miR, but there is also compelling evidence that the mature levels of these three miRs are regulated individually as well. Transcriptional control of the human miR-183 cluster has been shown to be regulated by both transcription and DNA methylation. Several transcription factors with established roles in embryogenesis and carcinogenesis, such as β-catenin/ TCF/LEF, p53 and TGF-β, have been shown to bind a promoter located 1.5–5kb upstream of hsa-miR-183 (87,98,125,127,150,157,165,168–169) (summarized in Table 2). There are several CpG islands before the miR-183 TSS (3.5, 8 and10 kb) and epigenetic regulation by DNA methylation has been shown (Figure 1A) (170). Of note, miR-182, not miRs-96 or -183, was solely regulated by demethylation of a CpG island −10 kb upstream in a study by Liu *et al*., in human melanoma cell lines (171).

**Table 2.** Transcriptional regulators of the miR-183 cluster

Transcriptional regulator	Model system	Approx. binding site location	Method	Direction of regulation	Ref.
MYCN	Human neuroblastoma cell lines	∼5 kb upstream of hsa-miR-183	Chip-Seq	↓	(168)
HDAC2	Human neuroblastoma cell lines	∼5 kb upstream of hsa-miR-183	Chip-Seq	↓	(168)
SREBP2	Murine liver	∼4 kb upstream of mmu-miR-183	ChIP-Seq and luciferase	↑	(157)
cMAF	Murine liver	∼56 kb upstream of mmu-miR-183	*In silico*, FACS and RT-PCR	↑	(165)
SP1	Lung cancer cell lines	∼500 bp upstream of hsa-miR-182	Luciferase and ChIP	↑	(98)
ZEB1	Common cancer cell lines	∼3.8 kb and ∼5.1 kb upstream of hsa-miR-183	Luciferase and ChIP	↓	(168)
β-CATENIN/ TCF4/LEF	Human gastric cancer cell line	6–8 kb upstream of hsa-miR-183	ChIP	↑	(127)
EVI1	Human pancreatic cancer cell lines	∼6 kb upstream of hsa-miR-96	ChIP	↓	(125)
p53	Primary human mammary epithelial cells	∼1.6 kb upstream of hsa-miR-183	ChIP	↑	(150)
TGFβ (via SMAD2 / SMAD4)	Human glioma cells	∼11.5 kb upstream of hsa-miR-182	ChIP	↑	(87)
FOXP3	Lymphoma cells	∼400 bp upstream of hsa-miR-183	ChIP and luciferase	↑	(169)

There are other reports of independent regulation of miRs -183, -96 and -182 levels, and the unusually long 4.2 kb intergenic sequence between miRs-96 and -182 may be critical to regulation of mature miR expression. In the human DNA sequence, there is a region of high genomic conservation ∼2 kb upstream of miR-182, which is indicative of a secondary TSS for miR-182 (shown in the UCSD Genome Browser in Figure 1A). However, two secondary TSS in the miR-96 and -182 intergenic region have been described in the literature and neither of them are in this area of conservation (98,132). A Sp1-regulated secondary TSS 500 base pairs upstream of miR-182 was characterized in lung cancer cells (98). In murine soft tissue sarcomas, another secondary TSS ∼140 bp upstream of miR-182 was identified (132), though this sequence is not present the human region of the miR-183 cluster. miR-96 was reported to be negatively regulated by the transcription factor EVI1 in pancreatic cancer via a putative binding site 6–7 kb upstream of its sequence (125), although miRs-182 and -183 were not mentioned in this study. Given the limited number of current reports about independent regulation of the miR-183 family, further studies are needed to characterize independent regulation of the cluster members including transcriptional, post-transcriptional processing, miR modifications and/or miR decay.

## CONCLUSIONS

Normally the expression of the miR-183 cluster is highly specific to the sensory organs and is necessary for sensory development and circadian rhythm, which are crucial conserved evolutionary features observed in the animal kingdom (172,173). Perhaps it is this role in development that has led to 600 million years of conservation. However, dysregulation of the miR-183 family expression occurs in disorders unrelated to sensory organs. The high expression of these miRs in disease may be permissive or contribute to the altered post-transcriptional landscape in cancer, autoimmune and neurological disorders. Moreover, the individual miR-183 family members cooperate to regulate multiple components of both normal and disease pathways of sensory development, metabolism, apoptosis, DNA repair, metal homeostasis, immune system and circadian rhythm. Coming full circle, these miRs are also regulated by key transcriptional factors that control the above mentioned processes.

Several questions remain about the regulation and involvement of the miR-183 cluster in development and disease. Though there are promoter elements upstream of the miR-183 cluster, a secondary TSS in the miR-96 and -182 intergenic region may also be important to aberrant expression of the miRs in disease (98,132).

Another element that ties the miR-183 cluster to development and disease is that the miR-183 cluster expression can be regulated by β-catenin (127,174). β-Catenin is activated downstream of WNT signaling and is an evolutionary conserved transcription factor that is implicated in embryonic and organ developmental regulation (127,174–180). Based on this recent data, the miR-183 cluster is likely involved in certain aspects of embryonic development under the regulatory control of β-catenin. Though there are several reports of miR-183 family member expression in mouse, zebra fish, and even human embryonic stem cells, very little is known about its functional role in embryonic development outside the sensory organs. The miR-183 family null mouse has provided some insight into the role of these miRs in development, but there was not an overt disease phenotype. Perhaps, the short and controlled lifespan of laboratory mice does not permit these age-related diseases to emerge, and it is likely that abnormal expression of these miRs would have more pronounced phenotypes in humans. As well, better understanding of the half-life and stability of the pri-miR, pre-miRs and mature miRs would help provide insight into the cluster's organ-specific functions in various cellular environments.

Although not detailed in this review, there is growing evidence that these miRs may also serve as disease biomarkers and that certain SNPs, which are located near or within the miR-183 cluster, are associated with various diseases (86,90,100,109,181–184). However, further studies are needed to determine whether these SNPs are functionally significant to disease pathology. Since the miR-183 family has been implicated in mediating the sensitivity of various chemotherapies as described in section 5.2, additional studies are warranted to study this cluster as a biomarker of chemotherapeutic effectiveness.

In closing, the body of literature reinforces the concept that the highly conserved miR-183 cluster is a critical player in biological processes. Though each miR of the family possesses its own unique set of targets, coordinated expression of the cluster allows the miR-183 family to co-regulate multiple pathways vital to both development and disease.

## Supplementary Material

SUPPLEMENTARY DATA
