# Supplementary material for: The microRNA-183 cluster: the family that plays together stays together
Source: Nucleic Acids Res. 2015 Jul 13;43(15):7173–88. doi: 10.1093/nar/gkv703 (PMC4551935; doi:10.1093/nar/gkv703)
Supplement: SUPPLEMENTARY DATA [file supp_43_15_7173__index.html]

The microRNA-183 cluster: the family that plays together stays together — The microRNA-183 cluster: the family that plays together stays together — The microRNA-183 cluster: the family that plays together stays together — SUPPLEMENTARY DATA 

# The microRNA-183 cluster: the family that plays together stays together

## SUPPLEMENTARY DATA

- SUPPLEMENTARY DATA
- SUPPLEMENTARY DATA
