# Supplementary figures and images for: The microRNA-183 cluster: the family that plays together stays together
Source: Nucleic Acids Res. 2015 Jul 13;43(15):7173–88. doi: 10.1093/nar/gkv703 (PMC4551935; doi:10.1093/nar/gkv703)

## Slide 1
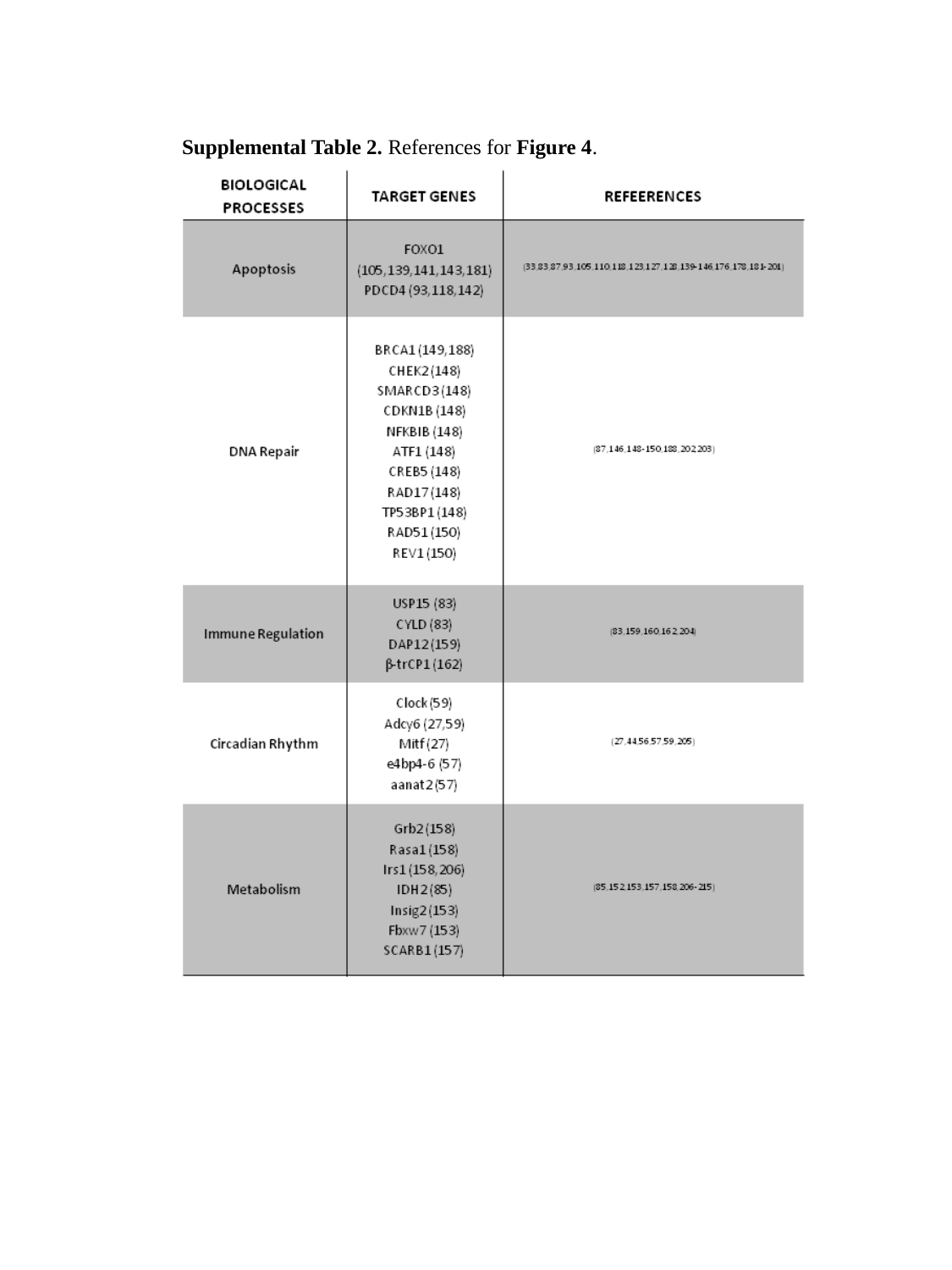

Supplemental Table 2. References for Figure 4.

Supplement: SUPPLEMENTARY DATA [file supp_gkv703_nar-01280-survey-d-2015-File002.pptx]
